# Promoting the use of the PRECISE score for prostate MRI during active surveillance: results from the ESOR Nicholas Gourtsoyiannis teaching fellowship

**DOI:** 10.1186/s13244-022-01252-1

**Published:** 2022-07-07

**Authors:** Francesco Giganti, Laurene Aupin, Camille Thoumin, Ingrid Faouzi, Hippolyte Monnier, Matthieu Fontaine, Alexandre Navidi, Paul-Gydéon Ritvo, Valentin Ong, Cecile Chung, Imen Bibi, Raphaële Lehrer, Nicolas Hermieu, Eric Barret, Alessandro Ambrosi, Veeru Kasivisvanathan, Mark Emberton, Clare Allen, Alex Kirkham, Caroline M. Moore, Raphaële Renard-Penna

**Affiliations:** 1 Department of Radiology, University College London Hospital NHS Foundation Trust, London, UK; 2 Division of Surgery and Interventional Science, University College London, 3rd Floor, Charles Bell House, 43-45 Foley St., London, W1W 7TS UK; 3 AP-HP, Radiology, Pitié-Salpêtrière Hospital, Sorbonne University, Paris, France; 4 Department of Urology, Institut Mutualiste Montsouris, Paris, France; 5 School of Medicine, Vita-Salute San Raffaele University, Milan, Italy; 6 Department of Urology, University College London Hospital NHS Foundation Trust, London, UK

**Keywords:** Urogenital neoplasms, Prostatic neoplasms, Magnetic resonance imaging, Biopsy

## Abstract

**Objectives:**

The PRECISE criteria for serial multiparametric magnetic resonance imaging (MRI) of the prostate during active surveillance recommend the use of a dedicated scoring system (PRECISE score) to assess the likelihood of clinically significant radiological change. This pilot study assesses the effect of an interactive teaching course on prostate MRI during active surveillance in assessing radiological change in serial imaging.

**Methods:**

Eleven radiology fellows and registrars with different experience in prostate MRI reading participated in a dedicated teaching course where they initially evaluated radiological change (based on their previous training in prostate MRI reading) independently in fifteen patients on active surveillance (baseline and follow-up scan), and then attended a lecture on the PRECISE score. The initial scans were reviewed for teaching purposes and afterwards the participants re-assessed the degree of radiological change in a new set of images (from fifteen different patients) applying the PRECISE score. Receiver operating characteristic analysis was performed. Confirmatory biopsies and PRECISE scores given in consensus by two radiologists (involved in the original draft of the PRECISE score) were the reference standard.

**Results:**

There was a significant improvement in the average area under the curve (AUC) for the assessment of radiological change from baseline (AUC: 0.60 [Confidence Intervals: 0.51–0.69] to post-teaching (AUC: 0.77 [0.70–0.84]). This was an improvement of 0.17 [0.016–0.28] (*p* = 0.004).

**Conclusions:**

A dedicated teaching course on the use of the PRECISE score improves the accuracy in the assessment of radiological change in serial MRI of the prostate.

## Key points

- A teaching course improves the accuracy in the assessment of radiological change in serial prostate MRI.
- Appropriate training should be delivered to radiologists to promote the use of the PRECISE score.
- Interactive courses could be used to certify radiologists reporting prostate MRI during active surveillance.

## Introduction

Active surveillance has been increasingly adopted as a management option in patients with low and favourable intermediate risk prostate cancer and a life expectancy of more than 10 years [1].

There has been increasing interest in the use of multiparametric magnetic resonance imaging (MRI) during active surveillance, and this technique has now become common place in active surveillance candidates’ selection due to its high negative predictive value for clinically significant disease [2].

There is also strong evidence to support the use of prostate MRI in patients with an initial biopsy suitable for active surveillance, and to target any lesions seen on imaging, often in conjunction with a confirmatory systematic biopsy [3].

The Prostate Cancer Radiological Estimation of Change in Sequential Evaluation (PRECISE) recommendations [4] represents the first standardised scoring system to report the likelihood of radiological change in serial imaging during active surveillance. At present, there is limited literature on the application of the PRECISE score in a clinical setting [5] and there is no formal investigation on the impact of a dedicated training course for radiologists in the assessment of radiological change in serial imaging.

The importance of reader training and experience in prostate MRI is evident from the literature [6, 7] and several studies on prostate MRI have shown considerable interobserver variability, providing evidence that reader experience is crucial for accurate reporting [8–10].

The Nicholas Gourtsoyiannis Teaching Fellowship, established by the European School of Radiology (ESOR), is aimed at radiologists who wish to enhance their teaching and training skills by delivering lectures and undertaking interactive workshops. For the year 2021, the fellowship has been awarded to two separate projects on prostate MRI.

We report here the results from the first project, which focussed on teaching the application of the PRECISE score on serial prostate MRI during active surveillance [4].

Our hypothesis was that dedicated training in reading serial prostate MRI during active surveillance by using the PRECISE score would significantly improve readers with different levels of experience in determining the degree of radiological change over time.

## Materials and methods

The PRECISE score consists of a 1-to-5 scale that includes repeated measurement of each lesion (if any), and attribution of a score for the likelihood of significant radiological change over time (Table 1). In detail, a PRECISE score of 1 or 2 means that there has been radiological regression, a PRECISE score of 3 entails that the MR findings are stable over time, while a PRECISE score of 4 or 5 refers to radiological progression. A dedicated reporting proforma (case report) should be used for each patient and for each MR scan, in order to collect the data in a systematic manner.

**Table 1:** Assessment of likelihood of radiological change on magnetic resonance imaging in patients on active surveillance for prostate cancer (PRECISE score)

PRECISE score	Assessment of likelihood of radiological progression
1	Resolution of previous features suspicious on MRI
2	Reduction in volume and/or conspicuity of features suspicious for prostate cancer
3	Stable MRI appearance: no new focal/diffuse lesions
4	Significant increase in size and/or conspicuity of features suspicious for prostate cancer
5	Definite radiologic stage progression (ECE, SV involvement, LN involvement, metastasis)

*MRI* magnetic resonance imaging, *ECE* extracapsular extension, *SV* seminal vesicle, *LN* lymph node

*Reprinted with permission from Moore CM, Giganti F, Albertsen P, *et al*. Reporting magnetic resonance imaging in men on active surveillance for prostate cancer: the PRECISE recommendations—a report of a European School of Oncology task force. Eur Urol 2017; 71:648–655*

The 2021 fellowship recipient (F.G.) is a Consultant Radiologist highly experienced in prostate MRI (i.e., reporting more than 2500 prostate MR scans per year) who had been actively involved in the draft and publication of the original PRECISE consensus paper [4].

**Fig. 1:**
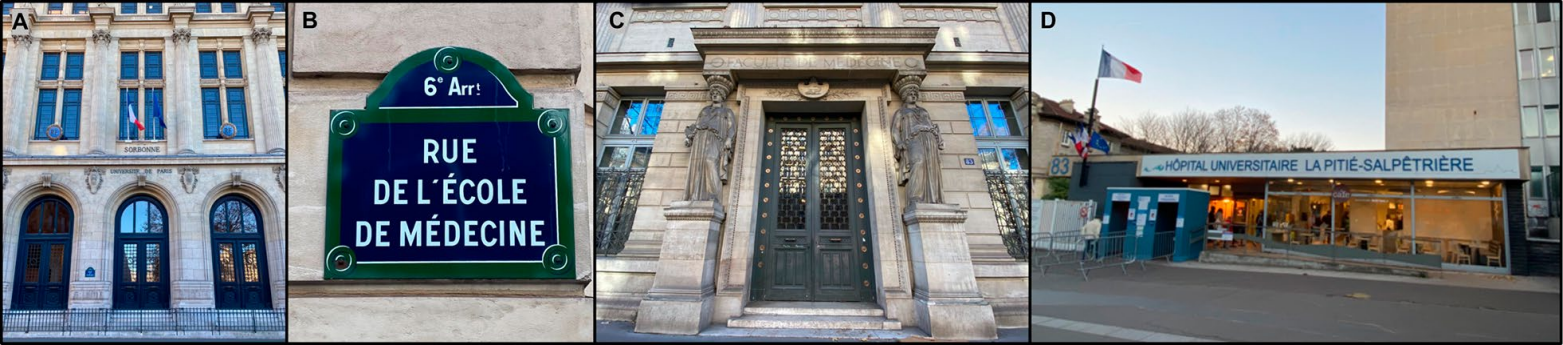
Sorbonne Université (**A–C**) and main entrance of Hôpital La Pitié-Salpêtrière in Paris (**D**), France. The images were taken during the teaching fellowship in November 2021

The first teaching fellowship took place at Sorbonne Université and Hôpital La Pitié-Salpêtrière in Paris (Fig. 1) between November 21 and November 28, 2021, in collaboration with one of the panellists (R.R.P.) who participated in the original PRECISE consensus meeting [4].

### Setting and participants

Eleven participants from the Radiology Department of Hôpital La Pitié-Salpêtrière with different levels of experience in prostate MRI reporting were invited to participate in this project, provided that they were unaware of the PRECISE scoring system and how to use it. They were aware that patients had prostate cancer, but they were blinded to all other clinical and laboratory data, including biopsy results and the original MR reports.

**Fig. 2:**
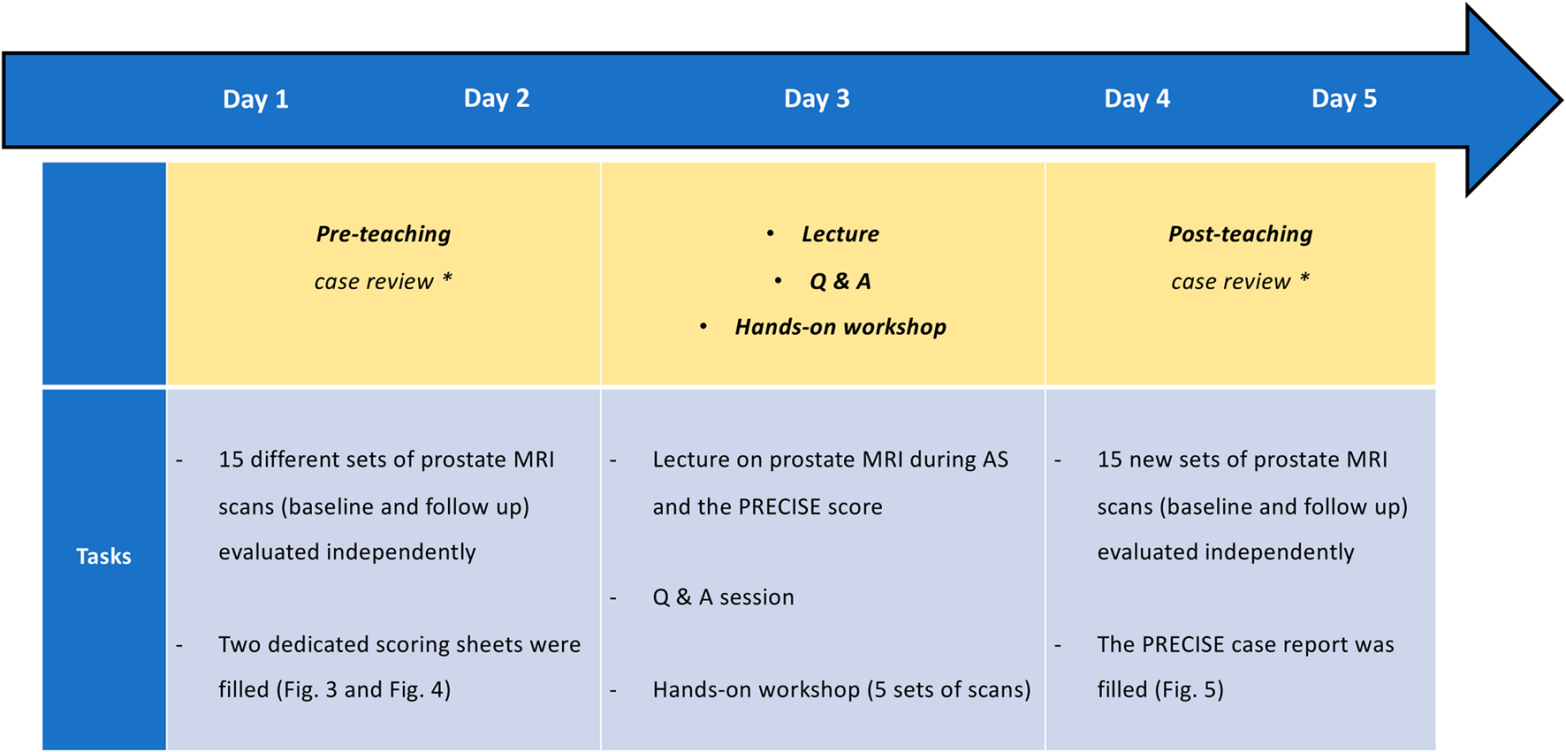
Chronologic framework of the teaching fellowship. *Readers were given up to two weeks (before / after the lecture and workshop) to review the images

Figure 2 shows the framework of each step of the teaching fellowship during the week.

**Fig. 3:**
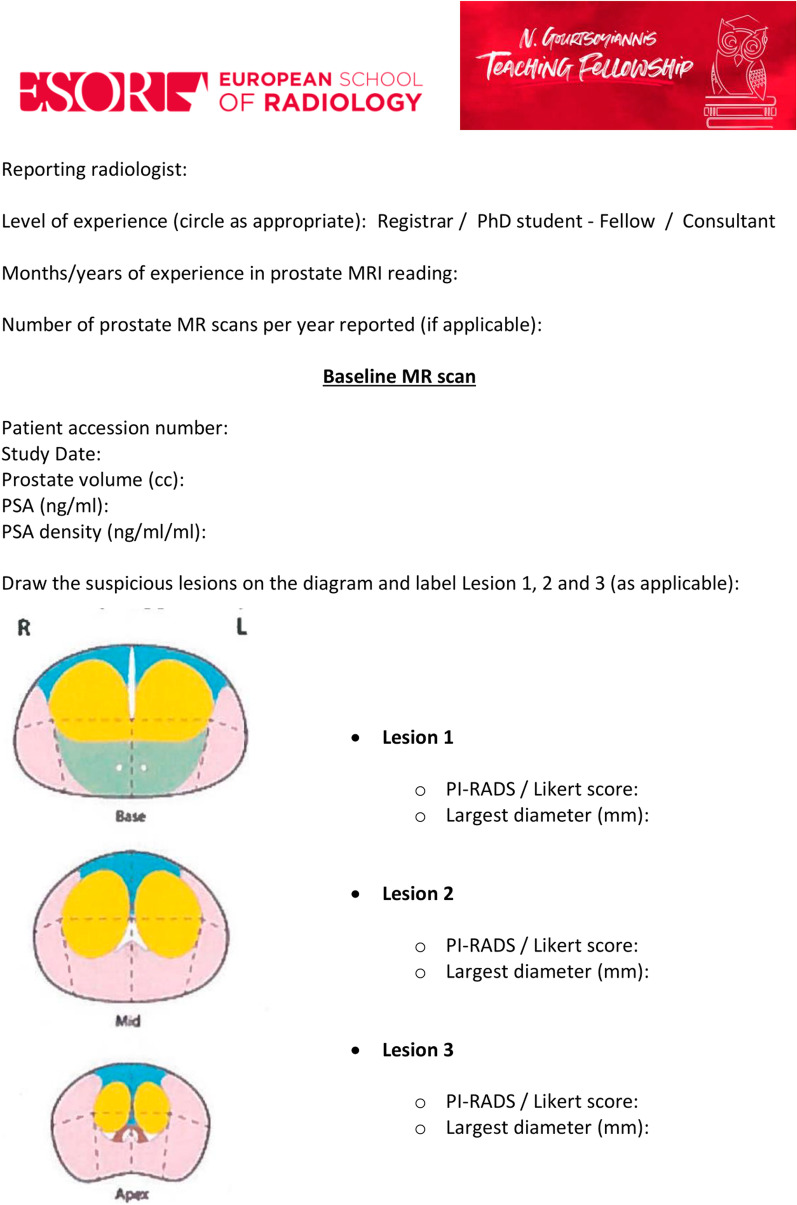
Scoring sheet for baseline scans (pre-teaching)

**Fig. 4:**
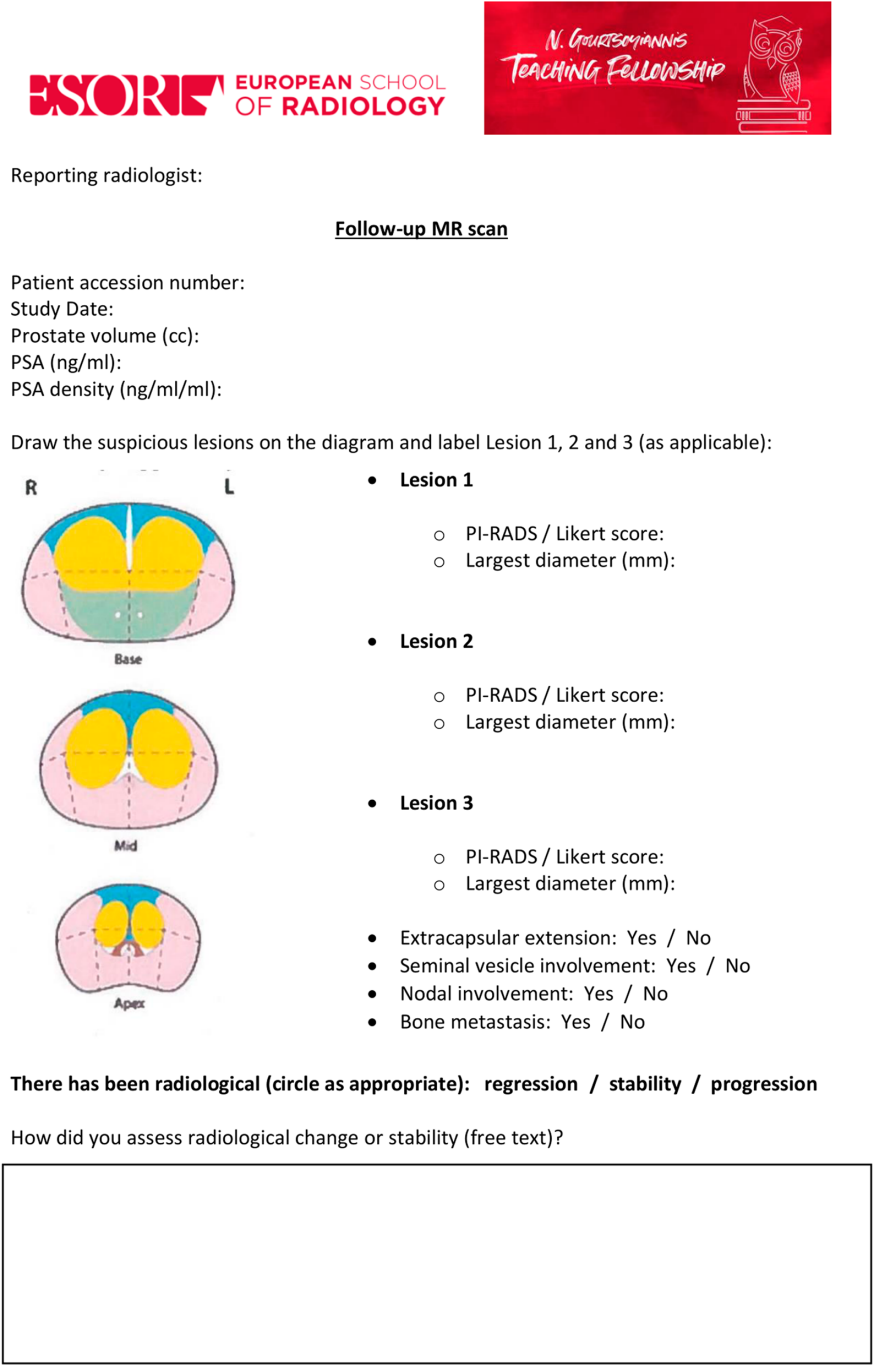
Scoring sheet for follow-up scans (pre-teaching)

- *Day 1* and *Day 2*: in the two weeks prior to the lecture, all participants were asked to go through the baseline and follow-up mpMRI scans of 15 patients independently and fill two scoring sheets (Figs. 3 and 4) that were specifically created for this project by the course director (F.G.). They were asked to draw up to three lesions and give a Prostate Imaging Reporting and Data System (PI-RADS) and/or Likert score together with the largest lesion diameter (mm) on the dominant sequence for the peripheral and transitional zone, as per PI-RADS v. 2.1 guidelines. [11] For the follow up scans the participants were also asked to evaluate the degree of radiological change (i.e., radiological regression, stability or progression) subjectively along with a narrative explanation on how radiological change was assessed. No further instructions or guidelines were given, and the participants relied only on their previous training in general radiology and prostate MRI reading. They were allowed to work at different speeds and spend as much time as they needed on the interpretation of the scans. All scoring sheets were returned before the lecture and the workshop (i.e., before the end of the ‘Day 2’ of the fellowship).
- *Day 3*: all participants attended the lecture and the workshop on the third day of the fellowship, and they were encouraged to ask questions at the end of the talk to improve their understanding of the subject.

### Lecture framework

The lecture, whose title was: “*MRI and active surveillance using the PRECISE recommendations: are we ready to take the next step?*” consisted of two separate modules, which were held on the same day:

- Module 1-*Prostate MRI and active surveillance: recommendations from national/international guidelines and overview of the current available literature*
- Module 2-*The PRECISE recommendations: standardising prostate MRI reporting during active surveillance*

All participants attended the lecture (1 h), which was followed by a Q&A session (30 min) in which they were encouraged to ask questions to improve their understanding of the subject.

### Workshop framework

**Fig. 5:**
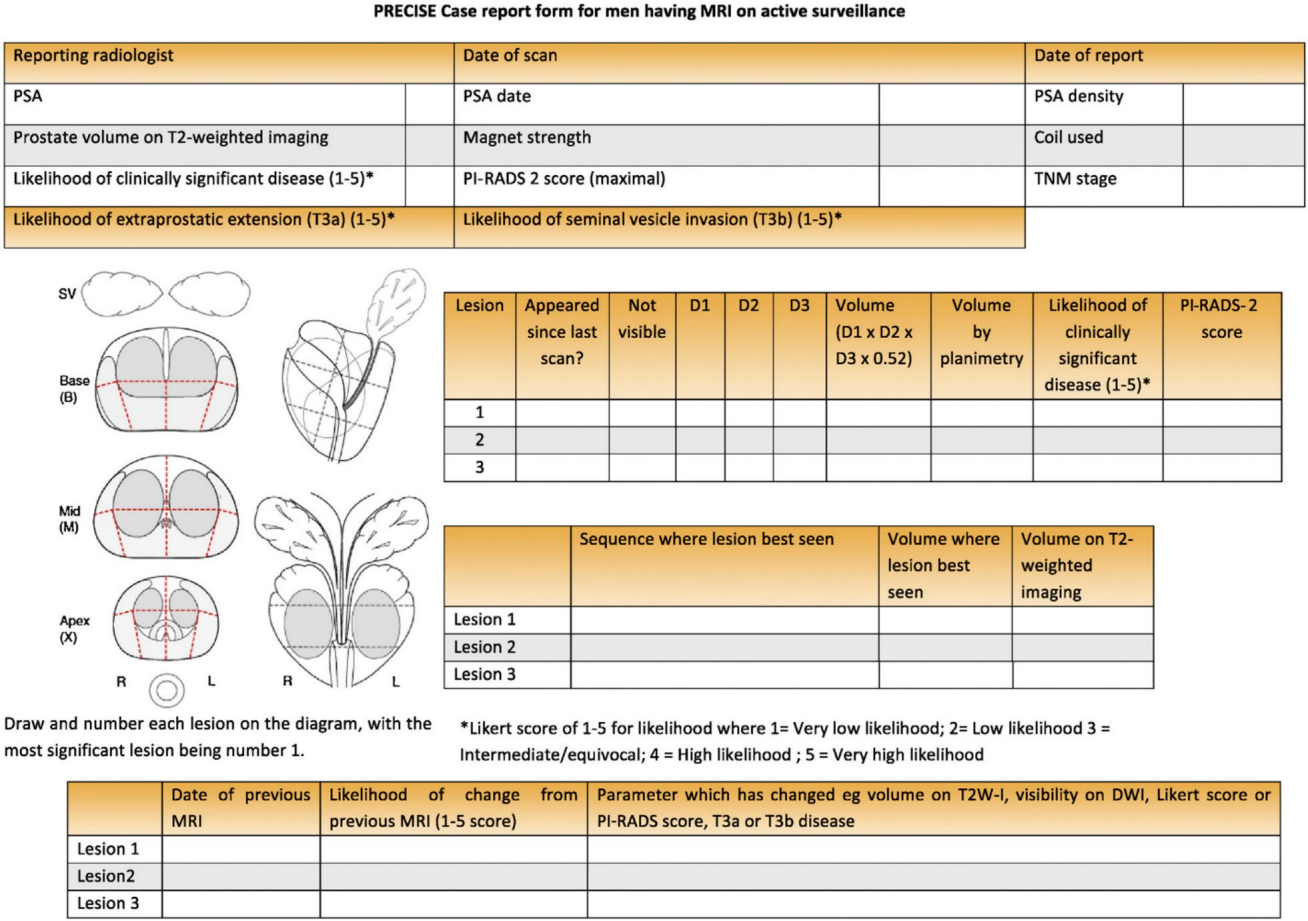
PRECISE case report form (post-teaching)

The workshop, whose title was: “*The PRECISE recommendations: from theory to practice*”, was carried out following the lecture and included hands-on training to familiarise the participants with the PRECISE scoring system. During the workshop, five different sets of scans that had been scored in the previous two weeks were reviewed and discussed collegially in light of the PRECISE recommendations. The participants were taught how to evaluate radiological change and assess the PRECISE score for each patient using the dedicated PRECISE case report form (Fig. 5).

- *Day 4* and *Day 5*: all participants were asked to evaluate a new set of images from 15 different patients (each of which included again a baseline and a follow-up scan) independently using the PRECISE case report form from the fourth day of the fellowship onwards and they were given up to two weeks after the lecture to return their scores. For this specific pilot study, it was agreed to collect only the largest diameter of each lesion on the dominant sequence, as this is allowed in the PRECISE recommendations [11].

The participants interpreted all the studies independently and at different times on dedicated picture archiving and communication system (PACS) stations.

### Clinical cohort and reference standard

This study was supported by the Health Data Centre of the Assistance Publique-Hôpitaux de Paris and approved by the Ethical and Scientific Board (IRB00011591). Consent was waived as these images were acquired as part of standard clinical care.

All scans used for this project were part of a prospectively maintained database on prostate MRI at Hôpital La Pitié-Salpêtrière and were randomly selected by a research study assistant not involved in the study prior to the start of the teaching fellowship from a list of eligible patients who met the following active surveillance criteria: (i) biopsy-confirmed prostate cancer [i.e., Gleason score ≤ 3 + 4 and prostate-specific antigen (PSA) ≤ 20 ng/ml]; (ii) two serial prostate MR scans.

The timing of MRI during active surveillance was based on both baseline risk (i.e., presence of a visible lesion on baseline imaging) and PSA or PSA density changes during follow-up. All patients had confirmatory biopsies (systematic and targeted when necessary) using either a transrectal or transperineal approach.

The two radiologists (F.G. and R.R.P.)—who were the principal investigators of this study—re-evaluated the images and assessed the PRECISE score in consensus for each scan before the teaching fellowship, blinded to the histological results from the confirmatory biopsies. Their PRECISE scores along with the histopathological results from confirmatory biopsies were used as the reference standard for the evaluation of the participants’ performance before and after the course.

### MRI protocol

All scans were performed at Hôpital La Pitié-Salpêtrière according to international guidelines. [11, 12] Two different scanners were used: one 1.5 T (Magnetom Aera, Siemens) and one 3 T system (Magnetom Skyra, Siemens), with a pelvic phased-array coil. The protocol comprised multiplanar T2-weighted, diffusion-weighted (including calculated high *b* sequences: 1400 s/mm^2^ and 2500 s/mm^2^) and dynamically contrast-enhanced acquisitions. All patients received an enema prior to the study, and 1 mg of Glucagon was administered intramuscularly prior to the examination.

### Statistical analysis

Continuous variables were summarised by medians and inter-quartile ranges.

The primary outcome was the change in the average area under the curve (AUC) for detection of radiological change (stratified by PRECISE 1-3 vs PRECISE 4-5) before and after teaching.

For the purpose of the analysis, the pre-teaching scores given in the dedicated scoring sheets (i.e., radiological regression/stability *vs* radiological progression) were dichotomised into PRECISE 1-3 vs PRECISE 4-5.

Receiver operating characteristic (ROC) curves were based on generalised linear mixed models with random effects on readers and cases. This approach generalises the Obuchowski-Rockette method and is described by Liu et al. [13, 14]

For each ROC curve and AUC value, 95% confidence intervals (CI) were computed by conditional bootstrap resampling (*B* = 50,000 samples).

Exact *p* values were computed by permutation methods to avoid any distributional assumption or asymptotic approximation and considered significant when < 0.05.

All statistical analyses were performed in R v. 4.1.3 (R Foundation for Statistical Computing, Vienna, Austria).

## Results

### Clinical cohort

A total of 60 anonymised scans from 30 patients were included in this study.

All scans were of optimal diagnostic quality [i.e., Prostate Imaging Quality (PI-QUAL) score ≥ 4] [15] and had been performed between September 2011 and October 2021.

Table 2 shows baseline and follow-up characteristics of the cohorts included in the study.

**Table 2:** Descriptive statistics of the two cohorts (pre- and post- course) included in the study

	Pre-course (*n* = 15)	Post-course (*n* = 15)
Age at diagnosis (years)	68 (59–72)	63 (59–70)
PSA at baseline MR (ng/ml)	7.75 (5.6–8.9)	6.04 (5.2–10.3)
Prostate volume at baseline MR (cc)	42.1 (37–56.2)	53.3 (32.7–80.3)
PSA density at baseline MR (ng/ml/ml)	0.16 (0.1–0.24)	0.13 (0.1–0.17)
*Gleason score at entry biopsy*		
3 + 3	14 [93]	10 [67]
3 + 4	1 [7]	5 [22]
*Gleason score at confirmatory biopsy*		
3 + 3	3 [20]	10 [67]
3 + 4	8 [53]	4 [26]
4 + 3	3 [20]	1 [7]
4 + 4	1 [7]	–
*Baseline highest PI-RADS score*		
2	1 [7]	2 [13]
3	6 [40]	6 [40]
4	8 [53]	7 [47]
5	–	–
*Follow up highest PI-RADS score*		
2	1 [7]	3 [20]
3	2 [13]	4 [26]
4	10 [67]	6 [40]
5	2 [13]	2 [14]
*PRECISE score*		
1	–	–
2	1 [7]	–
3	2 [13]	10 [67]
4	10 [67]	4 [26]
5	2 [13]	1 [7]

Data are medians and interquartile range (parentheses); percentages in brackets [%]

*PSA*: Prostate Specific Antigen; *MR*: Magnetic Resonance; *PI-RADS*: Prostate Imaging–Reporting And Data System; *PRECISE*: Prostate Cancer Radiological Estimation of Change in Sequential Evaluation

### Reference standard and PRECISE score

For each case (pre- and post-teaching), the results from confirmatory biopsy along with the PRECISE scores given by the two principal investigators before the course served as the reference standard.

There were 12/15 (80%) and 5/15 (33%) patients showing radiological progression (i.e. PRECISE score ≥ 4) in the pre- and post-teaching cohorts, respectively (Table 2).

### Accuracy in assessing radiological progression

**Fig. 6:**
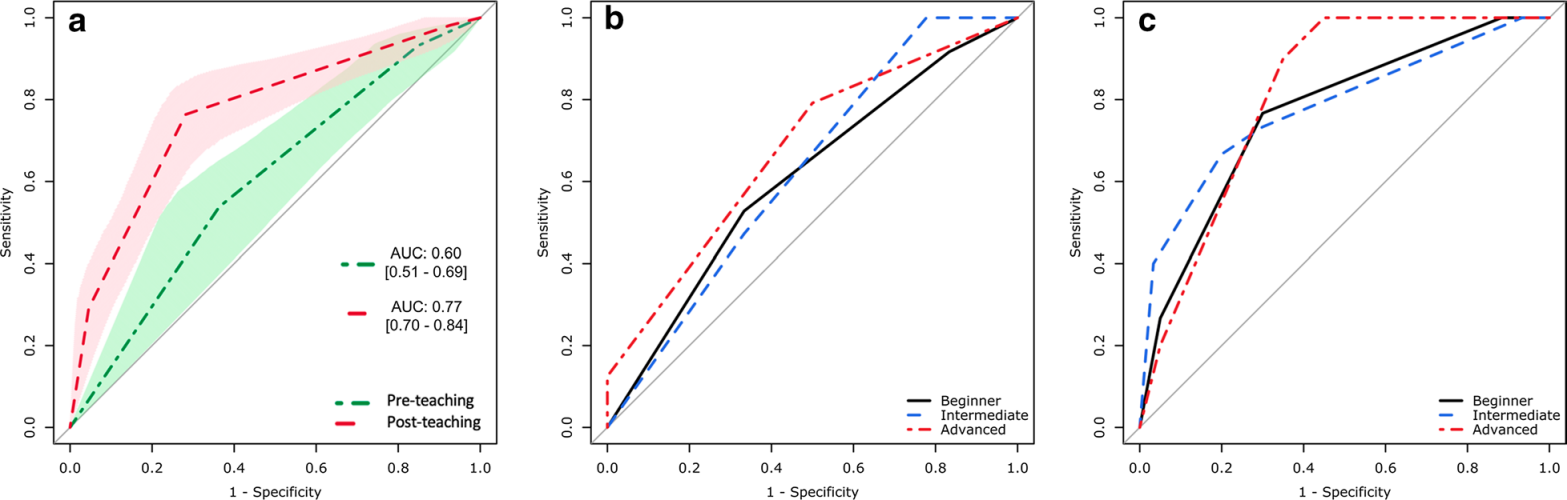
Average area under the curve (AUC) for the detection of radiological change in the pre-teaching (red, dash-dotted line) and post-teaching (green, dashed line) cohorts, with shaded areas and square brackets representing the 95% confidence intervals (**a**). AUCs for the detection of radiological change stratified by reader experience (as reported in Table 3) before (**b**) and after teaching (**c**)

There was a significant improvement in the average AUC for the assessment of radiological change from baseline (AUC: 0.60 [CI: 0.51–0.69]) to post-teaching (AUC: 0.77 [0.70–0.84]), an improvement of 0.17 [0.016–0.28] (*p* = 0.004). The ROC curves presented in Fig. 6a summarise the average accuracy levels in determining radiological change (stratified by PRECISE 1-3 vs PRECISE 4-5) on prostate MRI during active surveillance before and after the teaching course.

Table 3 reports the AUC in the assessment of radiological change stratified by reader experience before and after teaching. Interestingly, a nearly significant difference (*p* = 0.054) was observed for beginners (i.e. those participants who had reported < 50 prostate MR scans before the course). The ROC curves shown in Fig. 6b and 6c are a visual representation of these findings.

**Table 3:** Areas under the curve in the assessment of radiological change before and after teaching stratified by reader experience

Experience*	Pre-teaching	Post-teaching	*p*
Beginner (*n* = 6)	0.60 [0.47–0.73]	0.77 [0.67–0.85]	0.054
Intermediate (*n* = 3)	0.62 [0.43–0.81]	0.78 [0.63–0.91]	0.223
Advanced (*n* = 2)	0.67 [0.47–0.86]	0.82 [0.67–0.93]	0.257

^*^Experience was stratified as: beginner (< 50 prostate MR scans reported), intermediate (between 50 and 100 prostate MR scans reported) and advanced (> 100 prostate MR scans reported)

Number of participants in parentheses and 95% confidence intervals in brackets

Tables 4 and 5 show the pre- and post-course assessments given by each reader and according to the reference standard (including the parameters changed from baseline scan), respectively.

**Table 4:** Pre-course assessment given by each reader and according to the reference standard (including the parameters changed from baseline scan)

Pre-course	Reference standard		Reader 1	Reader 2	Reader 3	Reader 4	Reader 5	Reader 6	Reader 7	Reader 8	Reader 9	Reader 10	Reader 11
	PRECISE score	Parameters changed	PRECISE score*										
Case 1	4	Size	4	3	2	3	3	3	3	3	2	3	3
Case 2	4	Size	3	3	3	3	3	2	4	3	3	3	4
Case 3	4	Size	3	3	3	3	3	2	2	3	3	3	3
Case 4	4	Size and PI-RADS score	3	4	4	4	4	4	3	4	3	4	4
Case 5	5	Size, PI-RADS score and stage progression	4	4	4	4	4	4	4	3	4	4	4
Case 6	4	Size	3	3	4	4	4	4	4	4	3	3	3
Case 7	4	Size	3	4	4	4	4	4	4	4	4	4	4
Case 8	4	Size	3	3	4	3	4	4	4	4	3	2	4
Case 9	5	Size, PI-RADS score and stage progression	4	2	4	4	4	4	4	3	4	4	4
Case 10	4	Size and PI-RADS score	3	3	4	4	3	4	3	3	3	3	3
Case 11	3	–	3	3	3	2	3	3	3	2	3	3	3
Case 12	2	Size (decrease)	4	3	3	3	3	4	4	3	3	3	4
Case 13	4	Size and PI-RADS score	3	4	2	2	4	3	4	4	4	3	4
Case 14	3	–	4	4	4	2	4	2	4	2	4	4	4
Case 15	4	Size	4	4	4	4	4	4	4	3	4	4	4

*PRECISE*: Prostate Cancer Radiological Estimation of Change in Sequential Evaluation; *PI-RADS*: Prostate Imaging Reporting And Data System

* The initial scores given in the scoring sheets (i.e., radiological regression, stability or progression) corresponded to PRECISE 1-2, PRECISE 3 and PRECISE 4-5

**Table 5:** Post-course assessment given by each reader and according to the reference standard (including the parameters changed from baseline scan)

Post-course	Reference standard		Reader 1	Reader 2	Reader 3	Reader 4	Reader 5	Reader 6	Reader 7	Reader 8	Reader 9	Reader 10	Reader 11
	PRECISE score	Parameters changed	PRECISE score										
Case 1	3		3	1	2	1	1	2	3	2	4	2	4
Case 2	3		3	3	4	2	4	4	3	3	3	3	3
Case 3	3		3	3	3	4	1	4	2	3	4	3	4
Case 4	4	Size	4	4	4	3	5	5	4	4	4	4	4
Case 5	4	Size	5	4	4	3	4	4	3	4	4	4	5
Case 6	3		3	4	3	4	3	3	4	5	4	5	4
Case 7	4	Size	1	4	4	4	4	5	4	3	4	4	4
Case 8	3		2	3	4	3	3	4	4	3	3	3	3
Case 9	3		3	3	3	3	1	3	3	3	3	4	3
Case 10	5	Size, PI-RADS score and stage progression	3	4	5	3	5	3	5	3	5	3	5
Case 11	3		4	3	3	3	4	4	5	3	5	2	5
Case 12	3		3	3	4	3	3	3	3	3	3	3	3
Case 13	4	Size and PI-RADS score	3	4	2	3	5	4	4	4	3	4	5
Case 14	3		3	3	3	3	3	3	3	3	1	3	3
Case 15	3		3	3	4	3	4	3	3	4	3	4	3

*PRECISE*: Prostate Cancer Radiological Estimation of Change in Sequential Evaluation; *PI-RADS*: Prostate Imaging Reporting and Data System

## Discussion

This pilot study has shown that a dedicated teaching course on the use of the PRECISE score in serial MRI of the prostate during active surveillance is beneficial for radiologists with different levels of expertise.

We observed that the participants’ average accuracy in determining the degree of radiological change over time significantly increased after an interactive didactic lecture and a hands-on workshop.

It is recognised that experience and subspecialty training impact the diagnostic performance of prostate MRI, as experienced readers perform better than less experienced radiologists in the interpretation of the images [16].

Also, dedicated teaching courses have been shown to improve readers’ performances [17, 18].

The eleven readers who evaluated the degree of radiological change on serial prostate MRI had different levels of expertise, as reported in Table 3. It should be noted that it is difficult to express reader experience in prostate MRI, as pointed out in a recent consensus meeting [19].

In this pilot study, all participants were radiologists at different stages of their career, and we decided to use an arbitrary threshold of 50 prostate MR scans to differentiate between beginner and intermediate level, and a threshold of 100 scans to distinguish between intermediate and expert readers [20].

All participants achieved satisfactory accuracy levels and our overall goal has been successful.

A recent meta-analysis has shown that serial MRI of the prostate, not alone but in addition to other clinical factors and biomarkers, allow to reliably rule in and rule out prostate cancer progression. [5] In particular, it has been pointed out that the PRECISE score is currently the sole and most reliable tool to limit intrareader variability and standardise reporting of serial MRI of the prostate during active surveillance [5, 21].

In addition to this, prostate MRI may offer an opportunity to follow patients on active surveillance without the need of performing further biopsies in the absence of signs of radiological progression by means of the PRECISE score, although robust data from prospective studies are still needed before widespread adoption of MRI as a tool to replace repeat biopsies in this setting [5, 22].

It follows that dedicated teaching courses to disseminate the use of the PRECISE score represent a good opportunity for the widespread use of this scoring system, and we believe that the results of our pilot study are a first step in the right direction and should deserve confidence.

We acknowledge that there are some limitations in this pilot study. The first is the lack of patients showing radiological regression (i.e., PRECISE 1 and 2) in the post-teaching cohort. However, this was not chosen deliberately but it is rather the result of the randomisation process carried out by an independent research study assistant not involved in the study and with any sort of clinical background.

The second limitation is that all participants had already received (or were receiving at the time of the course) formal clinical training in Radiology, meaning that their skills in general MRI semiotics could have already been at an intermediate-to-high level. This, in addition to the small number of patients and scans included in this pilot study, might explain the reason why the AUC for the pre-course assessment was already higher than 0.6. Further studies on larger datasets with multiple serial scans (i.e., more than two) and including participants without training in MR reading (e.g., students in their final years of medical school or before starting proper training in Radiology) will be more informative.

In conclusion, although there is plenty of useful teaching material on prostate MRI for self-learning available online, the results from our pilot study reiterate the importance of dedicated hands-on training courses for the evaluation of radiological change on serial prostate MRI using the PRECISE score.

We believe that a combination of simultaneous lectures and practical workshops is a move in the right direction to train the radiological community, and we hope that our initial results from this teaching fellowship, along with those from the other experience, [23] could act as a source of inspiration for future applicants and represent fertile ground for further courses like this, not only related to prostate cancer imaging.

## Data Availability

Further information is available from the corresponding author on reasonable request.

## Abbreviations

AUC: Area Under the Curve
CI: Confidence Intervals
COVID-19: Coronavirus disease 19
ESOR: European School Of Radiology
mpMRI: Multiparametric Magnetic Resonance Imaging
PACS: Picture Archiving and Communication System
PI-QUAL: Prostate Imaging QUALity
PI-RADS: Prostate Imaging Reporting And Data System
PRECISE: Prostate cancer Radiological Estimation of Change In Sequential Evaluation
PSA: Prostate Specific Antigen
ROC: Receiver Operating Characteristic
